# Georgia Medical Association

**Published:** 1890-05

**Authors:** 


### GEORGIA MEDICAL ASSOCIATION.

---

The forty-first meeting of the Georgia State Medical Association was called to order promptly at 10:30, April 16, in L'Arioso Opera House in Brunswick, by Dr. J. S. Todd.

President J. B. S. Holmes being absent, Dr. R. O. Engram took the chair.

The meeting was formally opened with prayer by Rev. Goldman.

Mr. W. E. Kay delivered the address of welcome for the physicians of the city. Col. J. W. Bennett followed in an address in behalf of the city of Brunswick. The remarks of both were heartily enjoyed and applauded.

Dr. Eugene Foster responded to the address of welcome in his most happy manner.

Vice-President Engram followed in an address on Food in Health and Disease.

Then Dr. Foster, as chairman of the programme committee, read his report, and recommended that the reading of papers be limited to twenty minutes, and the discussing of papers to five minutes. After considerable discussion pro and con by Drs. Goodrich, Foster, Westmoreland, Benedict and Stockton, the report was adopted.

The next order of business was the announcement of the Board of Censors. The Censors were Drs. R. Battey, S. B.

Hawkins, M. H. O'Daniel, B. R. Doster and G. W. Mulligan. Drs. Battey and Hawkins not being present, Drs. A. W. Griggs and J. S. Todd were appointed in their place.

Seventeen applications for membership were received and, on the recommendation of the Censors, they were elected.

By a unanimous rising vote, the Secretary was instructed to telegraph Dr. J. B. S. Holmes the sympathy, confidence and continued love of the association.

Secretary Moore read his report and it was adopted.

Treasurer Goodrich asked for further time, as his trunk, in which his report was contained, had miscarried.

After the reading of letters from President Holmes, Drs. Hawkins, Cortelyou, Humphries, Wells and Mann, expressing their regrets for not being present, the body adjourned to meet Thursday, April 17th, at 9 a. m.

Wednesday afternoon was given up to social enjoyment, and every one seemed to have done so, if we can judge by the buzz of conversation, the laughter and the singing.

All the physicians and many of the citizens were on board the steamer Pope Catlin bound for St. Simons and a trip around the harbor.

The clam bake and oyster roast given at St. Simons seems to have been heartily enjoyed by all, judging by the way the bivalves disappeared.

Promptly at 9 o'clock Thursday morning the meeting was called to order by Dr. Engram, Vice-President.

The minutes of Wednesday's session were read and approved. By resolution of the society, Secretary Moore was instructed to furnish the Brunswick Times with a copy of Dr. Engram's paper on Food in Health and Disease.

Dr.V.O.Hardon read the first paper of the meeting entitled, Two Cases of Rare Ovarian Tumors. The paper was prepared in the Doctor's usual careful manner and was well received.

Aseptic vs. Antiseptic Surgery was the subject of a very able paper by Dr. S. C. Benedict, of Athens.

Dr. J. A. Dunwoody stated that he was in full accord with the author.

Dr. W. F. Westmoreland, Jr. said he believed that aseptic and antiseptic surgery had saved more lives than any other surgical measure, and that it was equally applicable to the palace and the hovel, also that antiseptic surgery lessened the pain following an operation.

Dr. J. F. Lancaster emphasized the idea that aseptic must precede as well as accompany the antiseptic.

Dr. J. McF. Gaston said he did not believe in powerful septic germicides, and that great harm was frequently done by the use of the bichloride of mercury, particularly in wounds capable of absorption. He recommended the use of a solution of sodium chloride.

Dr. Eugene Foster thinks that cleanliness is the most fruitful source of success in surgery, and that carbolic acid is the bane of surgery.

Dr. Benedict stated that in three years careful research, he had failed to find a pure case of death from the use of the bichloride, if it was properly used.

Dr. Doster stated that several years ago he had a student who after nine months study had only gotten to salts, and he thought that many others must have stopped there.

Dr. H. McHatton read an exceptionally able paper on Railroad Surgery.

Dr. Elliot opened the discussion of this paper, and spoke of the difference between a splint and the plaster bandage. Said he preferred the splint because the bandage did not allow for any swelling that might occur.

Dr. P. L. Hillsman said he was very much pleased with the views of the previous speakers, and called attention to a splint made of zinc oxide and glue, and also said he frequently used shellac varnish applied to flannel with much satisfaction.

Dr. W. F. Westmoreland, Jr., stated that he used plaster bandages to secure rest, that the splint and bandage were one in substance. He most frequently used the plaster bandage, and had very little trouble with swelling. It was his rule to operate at once if shock was not too severe.

The Surgery of the Past, Present and Future was the subject of the oration by Dr. W. F. Westmoreland, Jr., who handled the subject in a very pleasing, as well as able manner, and the Dr. was heartily applauded.

In the afternoon, the first paper was by Dr. W, L. Bullard, on a case of Ostia Sarcoma, with exhibition of patient.

Dr. K. P. Moore read a paper on The Female Urethra, a Source of Trouble Liable to be Overlooked in Our Gynecological Investigations. The subject was well handled and was well received.

Dr. J. G. Earnest led in the discussion, and said he fully agreed with the writer's views.

Dr. Hardon stated that he fully concurred with the views expressed in Dr. Moore's paper, but would drop a word of caution to doctors not to carry the operation too far, else serious effects might follow.

The Secretary then read a paper for Dr. A. C. Davidson, on A Unique Case of Senile Gangrene, Followed by a Bloodless Operation, without Tourniquet or Ligature. This was discussed by Drs. Westmoreland and Cotter.

Dr. McHatton read a paper for Dr. H. J. Williams, on The Importance of Microscopical and Clinical Analysis of Urine. This paper was ably discussed by Drs. Griggs, Todd, Duncan and Doster. After the appointment of the Nominating Committee, the meeting adjourned till Friday morning, at 9 o'clock.

Friday morning the meeting was called to order by Dr. Todd, in the absence of both president and vice presidents.

Dr. Cotter of Macon read the first paper. His subject being Recent Advances in Rhinology and Ophthalmology. Dr. A. W. Calhoun ably discussed this paper.

The Secretary then read a paper by Dr. R. O. Engram, on Stricture of the Male Urethra, and Some Forms of Neuroses, which was well discussed by Dr. Gaston and others.

Dr. J. A. Butts gave a short talk on the climate of Brunswick.

The officers for the ensuing year were then elected. They were, President, A. W. Griggs, of West Point; First Vice President, J. A Dunwoody, of Brunswick; Second Vice President, E. W. Lane, of Screven County.

The President then appointed the following delegates to the American Medical Association:

Drs. J. S. Todd, K. P. Moore, J. G. Hopkins, Wm. O'Daniel, Eugene Foster, A. G. Whitehead, T. M. McIntosh, George C.

Dugas, E. W. Lane, A. W. Calhoun, Virgil O. Hardon, George H. Stone, W. Duncan, J. A. Dunwoody, J. A. Butts, S. B. Hawkins, T. O. Powell, W. L. Heard, E. C. Goodreich, P. E. Cortelyou, J. G. Greene, J. B. S. Holmes, R. Battey, H. F. Campbell, J. F. Lancaster, R. O. Engram, J. M. Hall, DeSassure Ford, W. B. Tate, W. O. Dobbins, J. M. Kelly and J. McF. Gaston.

The meeting then adjourned to meet in Augusta, on the third Wednesday in April, 1891.

If the hospitality of the people of Brunswick is to be judged by the Banquet, they are certainly the most hospitable people in the Empire State of the South.

As good a banquet may have been given, but surely none better, and we believe that Thursday night, April 17, 1890, will long remain as a most enjoyable night in the minds of the members of the Georgia Medical Association. F. O. S.

